# Euglycemic Diabetic Ketoacidosis (DKA) With Sodium-Glucose Transport Protein 2 (SGLT-2) Inhibitors

**DOI:** 10.7759/cureus.79309

**Published:** 2025-02-19

**Authors:** Ahmed Malik, Eiman Ahmed, Sara Hamid, Mohammed Ahmed, Hossam Abdalla

**Affiliations:** 1 Acute Internal Medicine, University Hospitals of North Midlands NHS Trust, Royal Stoke University Hospital, Stoke-on-Trent, GBR; 2 Internal Medicine, University Hospitals of North Midlands NHS Trust, Royal Stoke University Hospital, Stoke-on-Trent, GBR; 3 Neurology, University Hospitals of North Midlands NHS Trust, Royal Stoke University Hospital, Stoke-on-Trent, GBR

**Keywords:** critical care, diabetes complications, euglycemic dka, metabolic acidosis, sglt-2 inhibitors

## Abstract

This case highlights a rare yet severe presentation of euglycemic diabetic ketoacidosis (DKA) in a male in his mid-eighties with type 2 diabetes mellitus managed on SGLT-2 inhibitors. Despite nearly normal blood glucose levels, he developed profound metabolic acidosis and elevated ketones, necessitating emergency intervention. Initial treatment with intravenous fluids and insulin led to temporary stabilization. Still, progressive metabolic derangement and multi-organ dysfunction resulted in a transition to palliative care, ultimately leading to his demise. This case underscores the need for heightened clinical awareness, early diagnosis, and cautious use of SGLT-2 inhibitors, particularly in elderly patients with multiple comorbidities.

## Introduction

Diabetic ketoacidosis (DKA) is a life-threatening metabolic emergency characterized by hyperglycemia, ketosis, and metabolic acidosis [1]. It is a well-recognized complication of insulin deficiency, commonly seen in individuals with type 1 diabetes mellitus but also occurring in those with type 2 diabetes mellitus (T2DM) under certain stress conditions [2]. Traditional DKA is typically defined by marked hyperglycemia (blood glucose >250 mg/dL or >13.9 mmol/L), a key diagnostic criterion [3]. However, an atypical and potentially underdiagnosed variant, euglycemic DKA, presents with significant ketosis and acidosis despite normal or only mildly elevated blood glucose levels (<250 mg/dL or <13.9 mmol/L). This atypical presentation can lead to diagnostic delays and an increased risk of poor clinical outcomes, underscoring the need for prompt recognition and treatment [1].

## Case presentation

An elderly male in his mid-eighties with a significant medical history, including T2DM, ischemic stroke, deep venous thrombosis, epilepsy, and benign prostatic hyperplasia, was brought to the emergency department due to acute respiratory distress. The day before his admission, he experienced mild fatigue but reported no significant symptoms. Upon arrival, he exhibited worsening shortness of breath and general distress, but no chest pain, fever, cough, hemoptysis, syncope, or collapse was noted.

His regular medications included warfarin, empagliflozin, metformin, phenytoin, and tamsulosin. During examination, the patient appeared drowsy and unwell. His vital signs were as follows: blood pressure at 170/92 mmHg, heart rate of 112 bpm, respiratory rate over 40 breaths per minute, oxygen saturation at 85% on 60% oxygen, and a temperature of 37.2°C. Chest auscultation revealed bibasal crackles, mottled skin on the abdomen and lower limbs, and minimal ankle edema. No focal neurological deficits were found.

Upon admission, the patient's condition was thoroughly investigated through laboratory tests. The results revealed severe metabolic acidosis, with a venous blood gas showing a pH of 6.96 and bicarbonate of 8.2 mmol/L, indicative of profound acidemia. Serum ketones were markedly elevated at 6 mmol/L despite a nearly normal glucose level of 11.4 mmol/L, confirming the diagnosis of euglycemic DKA. Additional findings included an elevated white cell count of 33.2 × 10⁹/L and significantly raised C-reactive protein at 411 mg/L, suggesting a systemic inflammatory response. The patient's B-type natriuretic peptide was also elevated at 639 pg/mL, indicating potential cardiac strain.

Table 1 summarizes the key laboratory findings, highlighting the profound metabolic acidosis, inflammatory markers, and metabolic derangements.

**Table 1:** Laboratory investigations

Investigation	Result	Normal reference range
Venous blood gas		
pH	6.96	7.35-7.45
Bicarbonate	8.2 mmol/L	22-28 mmol/L
Lactate	3.7 mmol/L	0.5-2.2 mmol/L
Glucose	11.4 mmol/L	3.9-5.6 mmol/L
Blood tests		
White cell count	33.2 ×10⁹/L	4.0-11.0 ×10⁹/L
C-reactive protein	411 mg/L	<5 mg/L
Creatinine	62 µmol/L	60-110 µmol/L
Estimated glomerular filtration rate	87 mL/min	>60 mL/min
Troponin	Normal	<0.01 ng/mL
B-type natriuretic peptide	639 pg/mL	<100 pg/mL

**Figure 1:**
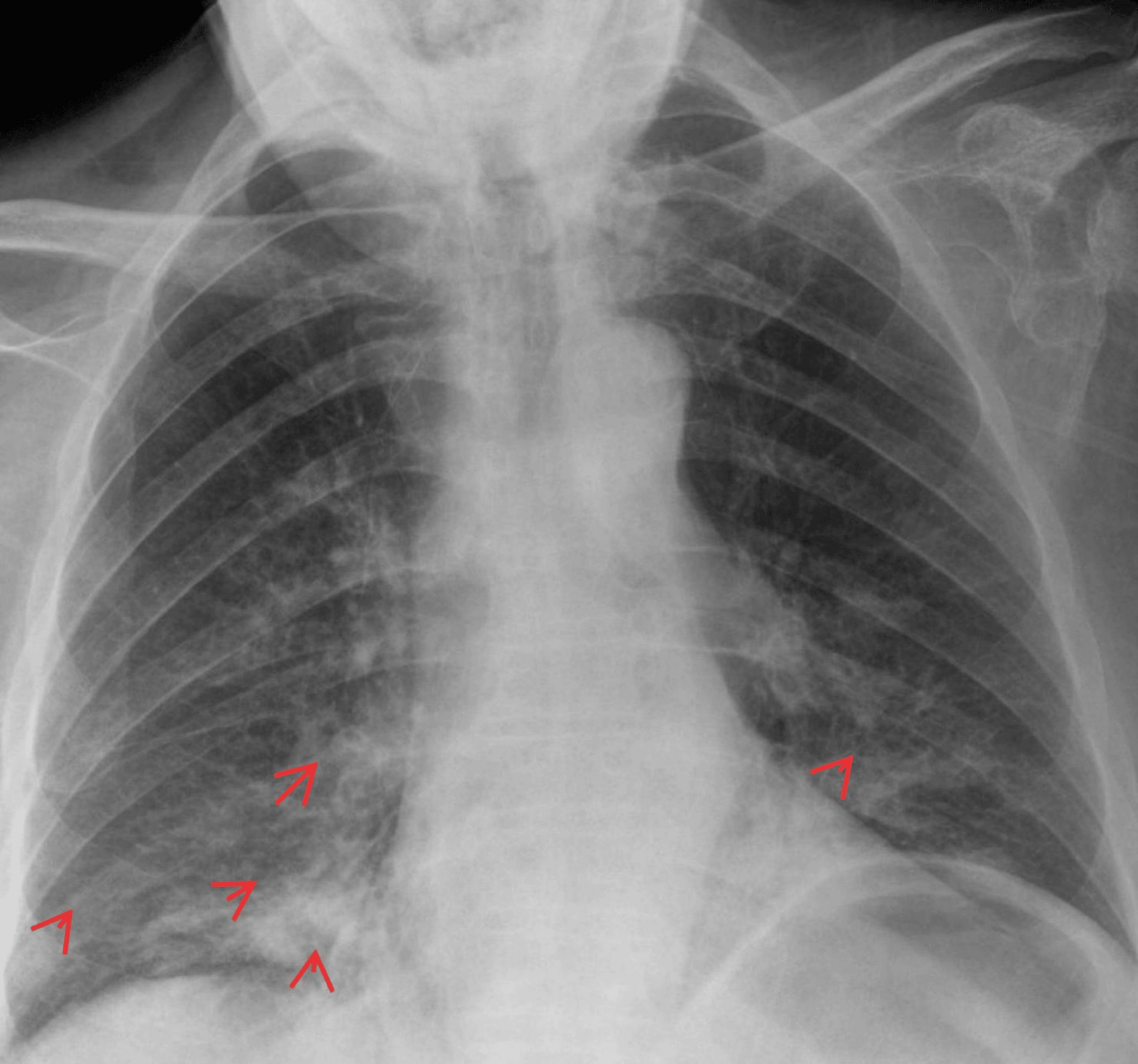
Chest X-ray showing bilateral lower zone opacities and areas of increased density (red arrows)

**Figure 2:**
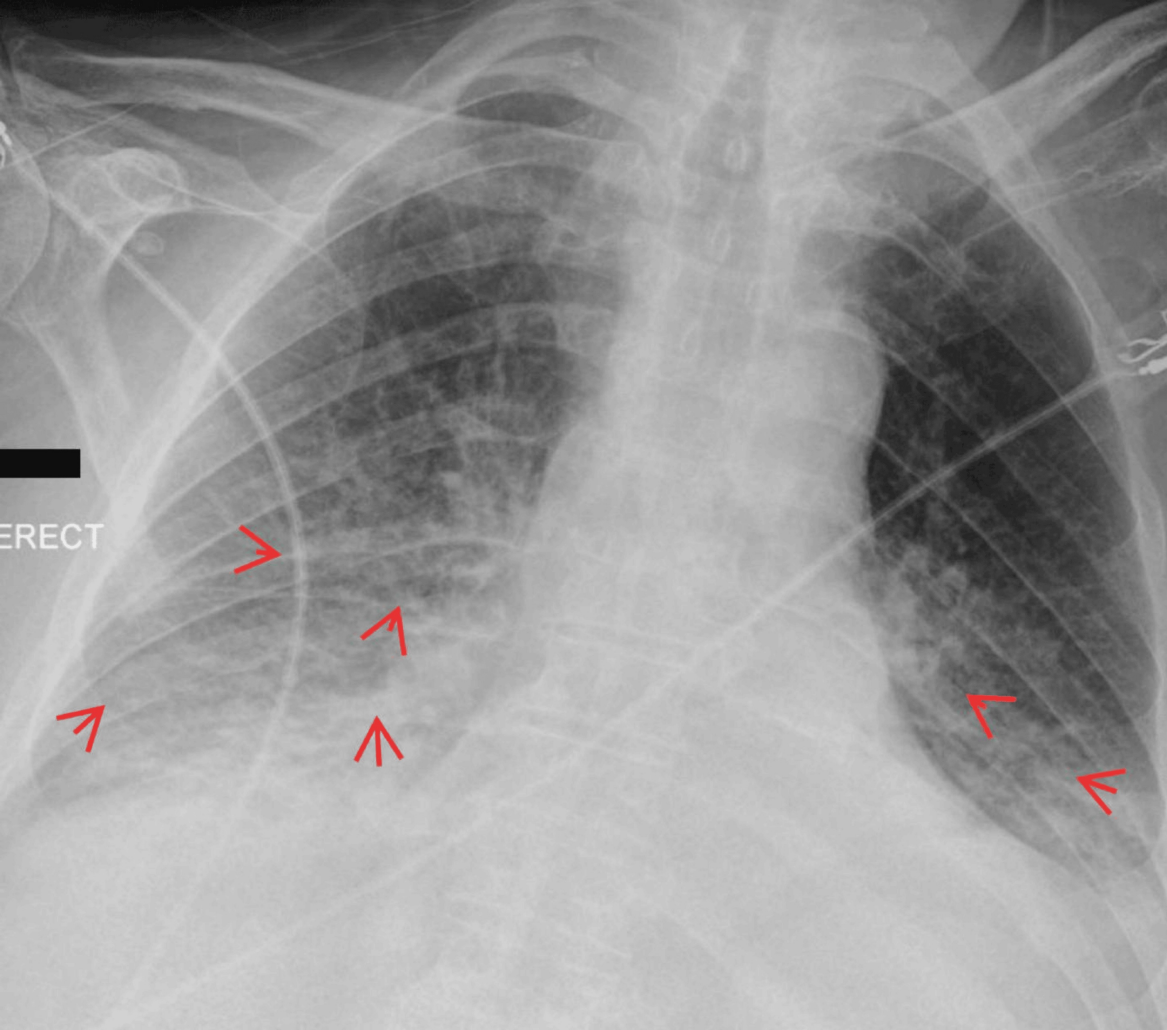
Chest X-ray indicating progressive bilateral opacifications (red arrows)

Figures 1-2 illustrate key diagnostic imaging findings that significantly contribute to the clinical assessment. Figure 1 presents a chest radiograph showing bilateral lower zone opacities, more pronounced on the right side, suggesting early infection. The highlighted region marks the area of increased density, raising suspicion of an infectious process. Figure 2 displays a repeated chest radiograph, where the highlighted region indicates progressive bilateral opacifications, consistent with evolving pneumonia rather than fluid overload. These radiographic findings are not just isolated observations but correlate directly with the patient's worsening respiratory status and systemic inflammatory response, providing a strong foundation for the clinical diagnosis.

An initial diagnosis of pneumonia with possible pulmonary edema was made. The patient received intravenous furosemide for suspected pulmonary edema, IV antibiotics for pneumonia, and discontinuation of empagliflozin and metformin due to metabolic acidosis concerns. Despite treatment, metabolic acidosis persisted. A repeat venous blood gas showed pH 6.98 and low bicarbonate levels, raising concerns for euglycemic DKA, confirmed with serum ketones at 6.0 mmol/L.

The diagnosis was updated to bronchopneumonia and SGLT-2 inhibitor-induced euglycemic DKA [1]. Treatment then focused on the DKA protocol, including IV fluids, insulin infusion, potassium replacement, and ongoing blood tests. The next day, the patient showed improvement: early warning signs improved from 11 to 4, oxygen needs decreased, and consciousness level rose, with pH at 7.32 and bicarbonate at 18 mmol/L.

However, later that evening, the patient deteriorated with increased oxygen needs, worsening respiratory distress, and hemodynamic instability. Due to his frailty and poor prognosis, the decision was made to transition to palliative care. The patient eventually passed away peacefully.

## Discussion

Euglycemic DKA is a rare but serious adverse effect of SGLT-2 inhibitors. Unlike typical DKA, which presents with profound hyperglycemia, euglycemic DKA is characterized by significant ketoacidosis despite normal or mildly elevated blood glucose levels. This atypical presentation increases the risk of misdiagnosis or delayed recognition, potentially leading to severe metabolic decompensation if not promptly treated [1].

Euglycemic DKA and SGLT-2 inhibitors

Euglycemic DKA has been increasingly associated with using SGLT-2 inhibitors, a class of oral antihyperglycemic agents approved for T2DM management. These medications promote renal glucose excretion, lowering blood glucose levels independent of insulin action. While SGLT-2 inhibitors offer significant benefits, including improved glycemic control, cardiovascular protection, and renal benefits, they also reduce insulin secretion and increase glucagon release, creating a metabolic environment favoring lipolysis and ketogenesis. This mechanism explains why patients taking SGLT-2 inhibitors can develop DKA without significant hyperglycemia, making early identification of euglycemic DKA particularly challenging [2].

Challenges in diagnosis and management

The nonspecific and insidious presentation of euglycemic DKA contributes to frequent misdiagnosis or delayed intervention. Symptoms such as nausea, vomiting, abdominal pain, fatigue, and respiratory distress are often mistaken for other conditions, such as sepsis, acute kidney injury, or lactic acidosis. Clinicians may overlook ketoacidosis in the absence of marked hyperglycemia, leading to inappropriate initial management. Triggers for euglycemic DKA include infection, dehydration, fasting, surgery, or reduced insulin therapy, all of which exacerbate ketone production and acid-base disturbances [3].

SGLT-2 inhibitor awareness

With the increasing use of SGLT-2 inhibitors for managing T2DM and their expanding cardiovascular and renal protection indications, clinicians must remain vigilant for potential adverse effects. These medications lower blood glucose levels by promoting urinary glucose excretion, leading to relative insulin deficiency. This insulin-deficient state and increased glucagon secretion promote lipolysis and ketogenesis, predisposing patients to ketoacidosis even when blood glucose remains within the normal range. Additionally, SGLT-2 inhibitors may mask the early hyperglycemic warning signs of DKA, delaying diagnosis [2].

Early recognition and diagnosis

Given the absence of marked hyperglycemia, euglycemic DKA may initially be mistaken for other causes of metabolic acidosis, such as sepsis, lactic acidosis, or renal failure. In this case, the patient presented with acute respiratory distress and metabolic acidosis, highlighting the urgent need for a high index of suspicion, especially in those taking SGLT-2 inhibitors. Key diagnostic indicators of euglycemic DKA include metabolic acidosis with low bicarbonate and an elevated anion gap, elevated serum ketones, and standard or only mildly elevated blood glucose, distinguishing it from typical DKA [3]. Early detection is critical, as delayed diagnosis can result in worsening acid-base imbalance, multi-organ dysfunction, and increased mortality risk, particularly in elderly or frail individuals.

Management considerations

Euglycemic DKA management follows standard DKA protocols but requires specific attention to the condition's nuances. Key treatment strategies include the immediate discontinuation of SGLT-2 inhibitors to halt further ketogenesis and aggressive intravenous fluid resuscitation to correct dehydration and improve renal clearance of ketones. Insulin therapy is essential to suppress lipolysis and promote glucose uptake, even if blood glucose levels appear normal. Frequent electrolyte monitoring and correction, particularly potassium, are necessary to prevent cardiac and neuromuscular complications. Additionally, identifying and treating underlying triggers, such as infections, dehydration, or recent reductions in insulin therapy, is crucial. Despite early initiation of therapy, the patient's condition deteriorated, ultimately requiring a transition to palliative care. This case reinforces the need for timely intervention and close monitoring, particularly in high-risk patients.

Risk factors for euglycemic DKA include specific patient populations at an increased risk of developing euglycemic DKA while on SGLT-2 inhibitors. These include elderly patients with multiple comorbidities who may have impaired compensatory mechanisms. Patients with intercurrent illnesses, such as infections or inflammatory conditions, can experience increased metabolic stress and ketone production. This leads to dehydration due to reduced oral intake, excessive diuresis, or gastrointestinal losses. Additionally, individuals with recent insulin dose reduction or discontinuation are at a heightened risk, as insulin deficiency is a key driver of ketogenesis. Postoperative patients or those undergoing prolonged fasting are particularly vulnerable, as prolonged fasting states exacerbate ketone production [3].

Clinical implications

Given the increasing use of SGLT-2 inhibitors, clinicians must carefully weigh the benefits of these agents against the potential risks, particularly in vulnerable populations. Patient education is not just crucial; it is a proactive measure that ensures individuals and caregivers recognize the warning signs, including nausea, vomiting, fatigue, and abdominal pain, in the absence of hyperglycemia. Preventive strategies should be emphasized, such as pausing SGLT-2 inhibitors during acute illness or fasting periods ("sick day rules") [3-5].

This case highlights the importance of awareness, early recognition, and prompt management of euglycemic DKA to prevent life-threatening complications. Future research is needed to define risk stratification better and develop guidelines for safer prescribing practices in high-risk patients [1].

## Conclusions

Euglycemic DKA is a rare yet serious complication of SGLT-2 inhibitors, occurring even when blood glucose is normal or only slightly elevated, often delaying diagnosis and treatment. This condition can lead to severe metabolic disturbances and poor outcomes, especially in elderly patients or those with multiple health conditions. Healthcare providers must remain vigilant and suspect euglycemic DKA in unexplained metabolic acidosis, even with normal glucose levels. Prompt action, including discontinuing the SGLT-2 inhibitor, administering IV fluids, insulin therapy, and correcting electrolytes, is crucial for better outcomes. Patient education is vital, as users should recognize DKA warning signs such as nausea, vomiting, abdominal pain, and altered mental status, particularly during fasting, illness, or dehydration. As SGLT-2 inhibitors become more widely used for blood sugar management and heart/kidney protection, this careful patient assessment and timely intervention are essential to reduce the risk of this potentially life-threatening condition, underscoring the urgency and importance of the healthcare provider's role.
